# Development and Efficacy Assessment of an Angle Sensor-Integrated Upper Limb Exoskeleton System for Autonomous Rehabilitation Training

**DOI:** 10.3390/s25133984

**Published:** 2025-06-26

**Authors:** Linshuai Zhang, Xin Tian, Yaqi Fan, Tao Jiang, Shuoxin Gu, Lin Xu

**Affiliations:** 1 School of Intelligent Medicine, Chengdu University of Traditional Chinese Medicine, Chengdu 611137, China; zhanglinshuai@cdutcm.edu.cn (L.Z.); tianxin20040522@163.com (X.T.); fanyaqi2025@163.com (Y.F.); xulin@cdutcm.edu.cn (L.X.); 2 School of Automation, Chengdu University of Information Technology, Chengdu 610225, China; gsx@cuit.edu.cn; 3 Engineering Research Center for Artificial Intelligence in Traditional Chinese Medicine, Chengdu 611137, China; 4 International Joint Research Center of Robotics and Intelligence System, Chengdu University of Information Technology, Chengdu 610225, China

**Keywords:** upper limb exoskeleton, angle sensor, self-rehabilitation training, four degrees of freedom

## Abstract

In this study, we propose a rehabilitation training system that incorporates active and passive rehabilitation modes to enhance the convenience, efficacy, and safety of rehabilitation training for patients with upper limb hemiplegia. This system facilitates elbow flexion and extension as well as wrist and palm flexion and extension. The experimental results demonstrate that the exoskeleton robot on the affected limb exhibits a rapid response and maintains a highly synchronized movement with the unaffected upper limb equipped with an angle sensor, preserving stability and coordination throughout the movement process without significant delay affecting the overall motion. When the movement of the unaffected upper limb exceeds the predetermined angle threshold, the affected limb promptly initiates a protective mechanism to maintain its current posture. Upon equalization of the angles between the two limbs, the affected limb resumes synchronized movement, thereby ensuring the safety of the rehabilitation training. This research provides some insights into the functional improvements of safe and reliable upper limb exoskeleton rehabilitation training systems.

## 1. Introduction

Stroke claims the lives of at least one in five individuals in China [1]. It is the primary cause of adult disability [2]. Most stroke survivors experience long-lasting functional impairments, with motor dysfunction being the most prevalent [3,4]. The cornerstone of treatment for these disabilities is rehabilitation, a process that allows stroke patients to reacquire optimal limb use and regain self-sufficiency [5]. Supplementary therapy may enhance post-stroke outcomes. Self-rehabilitation serves as a valuable method for extending the rehabilitation duration. Typically, mechanized systems are employed to prolong the motor training time. The main objective was to evaluate the impact of self-rehabilitation using a mechanized device compared with control self-exercise on upper extremity impairment in stroke patients [6,7,8]. Conventional rehabilitation programs depend on one-on-one treatment by physicians, and owing to high costs and low efficiency, upper-limb rehabilitation robots are gaining more attention. Traditional manipulation therapy faces numerous constraints, including therapist scarcity, prolonged and intensive collaboration between therapists and patients, subjective evaluation methods, and expensive rehabilitation training [9]. Consequently, there is a significant demand for advanced rehabilitation robots that are anticipated to assist patients in completing treatment more precisely, quantitatively, and individually [10,11,12]. Upper-limb exoskeleton robots are rapidly evolving and being implemented in various sectors, including industry and rehabilitation. Essentially, a wearable robot, the upper-limb exoskeleton’s following and strength assistance capabilities, can boost the wearer’s movement ability and restore function in patients with impaired or lost upper-limb mobility. Upper-limb rehabilitation robots can free professional rehabilitation physicians from repetitive, long-cycle physical work. They can establish tailored rehabilitation programs for patients to effectively improve upper limb proprioception and motor function [13], offering an advantage that doctors do not possess. The upper limb exoskeleton rehabilitation training system can substitute for medical staff in assisting patients with effective assisted rehabilitation training and enhancing their motor function [14,15].

For individuals with upper limb motor impairment, exercise assistance can be beneficial in restoring muscle strength and function, thereby enhancing their ability to perform daily self-care tasks [16]. In recent years, a growing body of research has demonstrated that exercise-assisted rehabilitation training can markedly improve the quality of life and motor capabilities of patients with upper-limb motor dysfunction. Consequently, there has been an increase in research focused on robotic devices designed to aid upper limb rehabilitation exercises [17,18,19,20,21]. Currently, extensive domestic and international studies have explored various driving methods for upper-limb exoskeleton robots, including motor drive, pull rope pulling drive, and pneumatic drive [22]. Each of these driving methods possesses unique characteristics and can accommodate different rehabilitation requirements. The motor drive, known for its precise motion control, is frequently employed in rehabilitation training. It offers advantages, such as a simple structure and lightweight design, making it suitable for compact exoskeleton equipment. The pneumatic drive generates force through air pressure changes, mimicking the flexible movement of muscles, which is appropriate for rehabilitation tasks that require high flexibility [23]. Typically, exoskeleton robots feature three to seven degrees of freedom, allowing for a more natural range of motion and multidirectional support for the upper limbs. These systems are equipped with high-precision internal sensors capable of capturing motion data that are challenging to obtain using conventional motion capture systems, with an accuracy comparable to that of professional motion capture equipment. This enables the harmonious exoskeleton robot to not only provide precise movement assistance to patients but also quantify and evaluate subtle movements during the rehabilitation process, offering more detailed and reliable data to support the adjustment and optimization of rehabilitation programs [24]. Through high-precision motion detection and data feedback, the exoskeleton system can adapt better to patients’ individualized rehabilitation needs, ultimately improving the effectiveness and efficiency of rehabilitation [25].

Rehabilitation robot equipment is typically classified into two types: exoskeletons and end-effector devices. Exoskeleton systems are designed to closely align human joints through their mechanical structure, enabling precise mapping of movements to the corresponding body joints. This feature provides excellent guidance and control during rehabilitation, making exoskeletons particularly beneficial for patients requiring extensive joint movement training to gradually restore limb function [26,27,28,29]. By contrast, end-effector devices primarily focus on endpoint exercises that target specific extremity movements. Their flexibility makes them more suitable for patients with spasticity [30]. End-effector equipment is especially effective in assisting with complex movements, such as shoulder rotation, elbow flexion, forearm rotation, and wrist bending. This makes them particularly useful for patients with motor disharmony resulting from nerve injury. Both exoskeletons and end-effector devices offer distinct advantages, providing effective support for various rehabilitation needs [31].

Our research led to the creation of a wearable exoskeleton system for rehabilitation training that was specifically engineered to facilitate synchronized movement between healthy and injured limbs. This system was designed to enhance limb coordination and motor function through self-directed exercises, offering an effective and sustainable rehabilitation platform. The core technology of the system enables coordinated movement of the unaffected and affected limbs through limb-guided motions. This approach allows patients to engage in independent training sessions without external assistance, leveraging the capabilities of their healthy limbs to support the rehabilitation of the injured limbs. A key advantage of this system is its self-directed training model that can deliver customized rehabilitation programs and reduce the need for external support. Additionally, the synchronous movement mechanism thoroughly stimulates the patients’ nervous and muscular systems, potentially enhancing the overall effectiveness of limb function recovery.

The main contributions of this study are as follows. First, in terms of hardware design, we developed a set of lightweight and highly adaptable wearable exoskeleton structures, which ensure wearing comfort and have good dynamic response performance, suitable for patients of different body types and rehabilitation stages. Second, in terms of the software system, we built a control platform integrating data acquisition, motion recognition, and feedback regulation, which realizes a user-friendly human-computer interaction interface and highly stable system operation, providing easy-to-operate solutions for clinical and home rehabilitation. Finally, we propose a synchronous motion control algorithm to track the trajectory of a healthy limb. Combined with real-time sensor data processing and a controllable angle threshold adjustment strategy, the dynamic guidance and action synchronization of healthy limbs to damaged limbs are realized, which effectively improves the precision of training and personalized level of rehabilitation. This study achieved multidimensional innovation in the system integration of wearable devices, adaptive synchronous control mechanisms, and personalized rehabilitation training strategies, providing a new path for efficient, autonomous, and scalable rehabilitation training for patients with motor dysfunction.

## 2. Materials and Methods

### 2.1. The Structure Design of the Upper Limb Exoskeleton Rehabilitation Training System

#### 2.1.1. Rehabilitation Posture and Degree of Freedom Design

**Figure 1:**
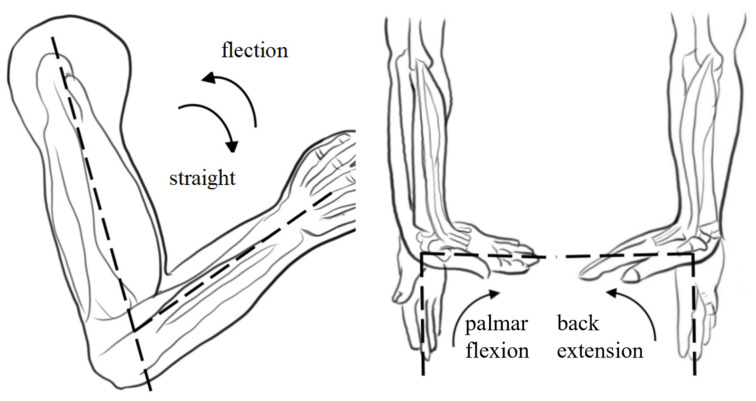
Schematic diagram of the elbow joint movement during upper limb rehabilitation training (note: The dotted line in the diagram is designed to more intuitively illustrate the normal range of motion for the elbow and wrist joints).

The human upper limb exhibits remarkable flexibility, primarily utilizing the shoulder, elbow, and wrist joints to perform various daily activities [32]. The primary motion range of the shoulder joint includes forward flexion/extension, abduction/adduction, and internal/external rotation. The elbow’s main movements include flexion/extension, posterior extension or hyperextension, and ulnar radial pronation/supination. The key wrist joint motions encompass palmar flexion/dorsal extension and ulnar/radial deviation. Our study primarily focused on upper limb rehabilitation exercises for the elbow joint with a normal range of motion (ROM) of 0–150° and a wrist joint with a normal range of motion (ROM) of −70–90° [33]. A schematic of the rehabilitation exercises is shown in Figure 1. Upper limb movement in humans is intricate and involves complex physiological structures such as muscles, bones, soft tissues, joints, and ligaments [34]. Implementing rehabilitation training for upper-limb exoskeletons across diverse scenarios presents significant challenges [35]. Consequently, this research emphasized localization and miniaturization, determining the exercise program of the upper limb exoskeleton rehabilitation training system based on human upper-limb movement characteristics.

#### 2.1.2. Mechanical Structure Design

The design quality of the mechanical structure significantly affects the system performance. When designing an upper limb exoskeleton rehabilitation training system, key considerations include training safety, human–machine interaction, structural lightness, and dexterity. To ensure patient safety during use, each movable joint incorporated a simple yet reliable mechanical limit structure. Additionally, to minimize patient burden, preference is given to compact, lightweight, and high-precision hardware that meets the rehabilitation requirements. To accommodate the varying physiological structures among users, a telescopic adjustment feature was integrated into the slide table of the elbow joint, enhancing system reusability.

**Figure 2:**
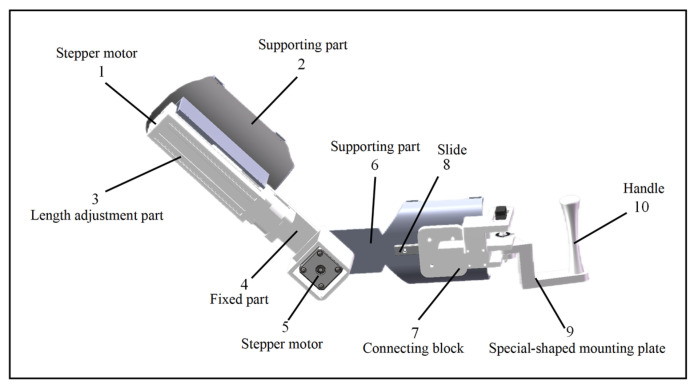
Structure diagram of the upper limb exoskeleton system.

The design parameters were based on the Chinese National Standard for Adult Body Size (GB/T 10000-2023) [36], which specifies an upper arm length of 251–350 mm and a forearm length of 185–243 mm. Taking into account the structural characteristics of the human body and the 2-degree-of-freedom rehabilitation posture, the design includes a 250 mm upper arm support with a 100 mm telescopic range, and a 190 mm forearm support with a 50 mm telescopic range. The structural design is illustrated in Figure 2.

### 2.2. Workspace Analysis of Exoskeleton

The workspace of an exoskeleton robot is one of the most important indicators for evaluating the flexibility of the exoskeleton. This refers to the collection of space points where the end effector of the exoskeleton robot can reach the space. At present, the simulation of the robot workspace generally establishes the forward kinematics equation of the robot, calculates the spatial coordinates of the end effector, and shows the set of spatial points that the end effector can reach in the coordinate diagram.

**Figure 3:**
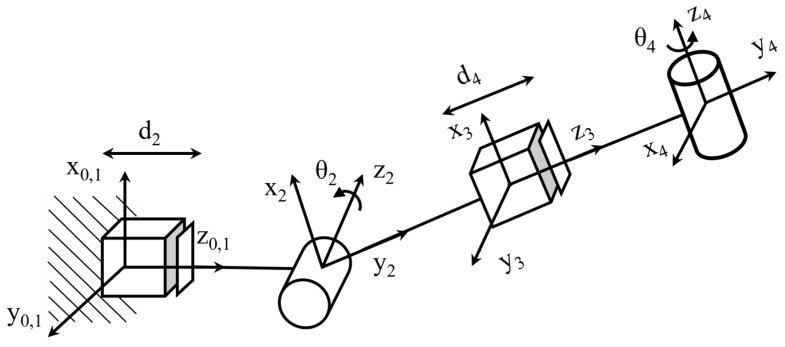
D-H coordinates of the upper limb exoskeleton system.

Using the D-H representation, the D-H coordinate system of the upper-limb rehabilitation exoskeleton robot was established. A four-degree-of-freedom upper-limb rehabilitation exoskeleton robot was designed in which the elbow contained one degree of freedom and the wrist contained one degree of freedom. The established link coordinate system is illustrated in Figure 3. Figure 3 shows the establishment of the model D-H coordinate system, including two telescopic joints and two rotational joints. *θ*_2_ and *θ*_4_ are the rotation angles of the rotational joints, the coordinate system of the shoulder fixed part is the *O*_0_ coordinate system, and the coordinate system of the end wrist part is the *O*_4_ coordinate system.

The D-H coordinates shown in Figure 3 and the D-H parameter table shown in Table 1 can be obtained according to the D-H coordinates shown in Figure 3.

The terminal manipulator pose of the upper-limb exoskeleton rehabilitation robot was obtained by translational rotation of the matrix. According to the D–H parameter table, the transformation matrix of each connecting rod can be expressed as

(1) $$T_{1}^{0}=\left(\begin{array}{cccc} 1 & 0 & 0 & 0\\ 0 & 1 & 0 & 0\\ 0 & 0 & 1 & 0\\ 0 & 0 & 0 & 1 \end{array}\right)\;T_{2}^{1}=\left(\begin{array}{cccc} c_{2} & 0 & s_{2} & 0\\ s_{2} & 0 & -c_{2} & 0\\ 0 & 1 & 0 & d_{2}\\ 0 & 0 & 0 & 1 \end{array}\right)$$

(2) $$T_{3}^{2}=\left(\begin{array}{cccc} 1 & 0 & 0 & 0\\ 0 & 0 & 1 & 0\\ 0 & -1 & 0 & 0\\ 0 & 0 & 0 & 1 \end{array}\right)\;T_{4}^{3}=\left(\begin{array}{cccc} c_{4} & 0 & -s_{4} & 0\\ s_{4} & 0 & c_{4} & 0\\ 0 & 1 & 0 & d_{4}\\ 0 & 0 & 0 & 1 \end{array}\right)$$

Then, the positive kinematics equation of the upper-limb exoskeleton rehabilitation training system is

(3) $$T_{4}^{0}=T_{1}^{0}T_{2}^{1}T_{3}^{2}T_{4}^{3}=\left(\begin{array}{cccc} c_{2}c_{4}-s_{2}s_{4} & 0 & -c_{2}s_{4}-s_{2}c_{4} & 0\\ s_{2}c_{4}+c_{2}s_{4} & 0 & -s_{2}s_{4}+c_{2}c_{4} & 0\\ 0 & 1 & 0 & d_{2}+d_{4}\\ 0 & 0 & 0 & 1 \end{array}\right)$$

where, $s_{i}=\sin\theta_{i}$, $c_{i}=\cos\theta_{i}$ in the above formula.

**Figure 4:**
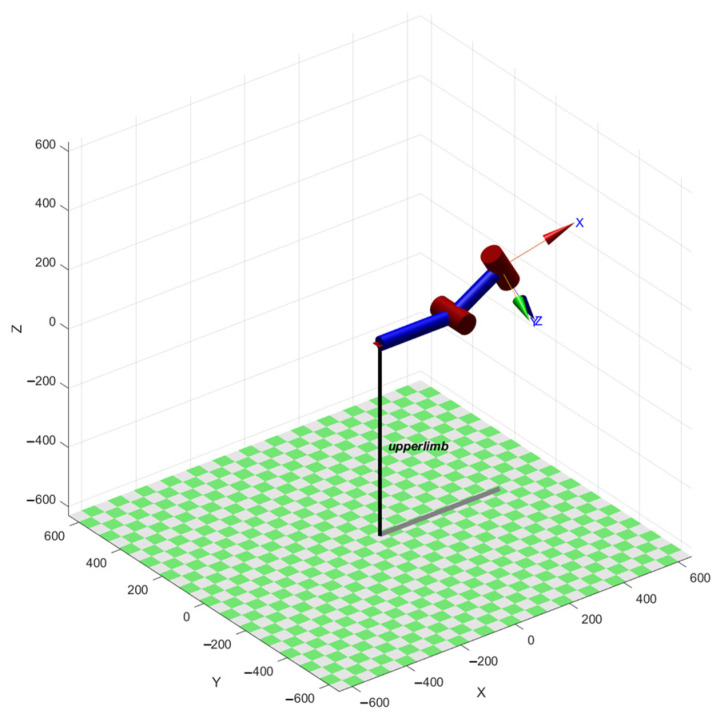
Simulation model of exoskeleton in simulation analysis software.

The upper-limb exoskeleton rehabilitation training system was modeled using a mathematical calculation and simulation analysis software. Let *d*_2_ = 270 mm, *θ*_2_ = pi/6, *d*_4_ = 200 mm, and *θ*_4_ = pi/9, as shown in Figure 4. After the comparison, it was found that the end position under the corresponding joint variables was consistent with the position and posture of the robotic arm, which verified the correctness of the kinematic model.

**Figure 5:**
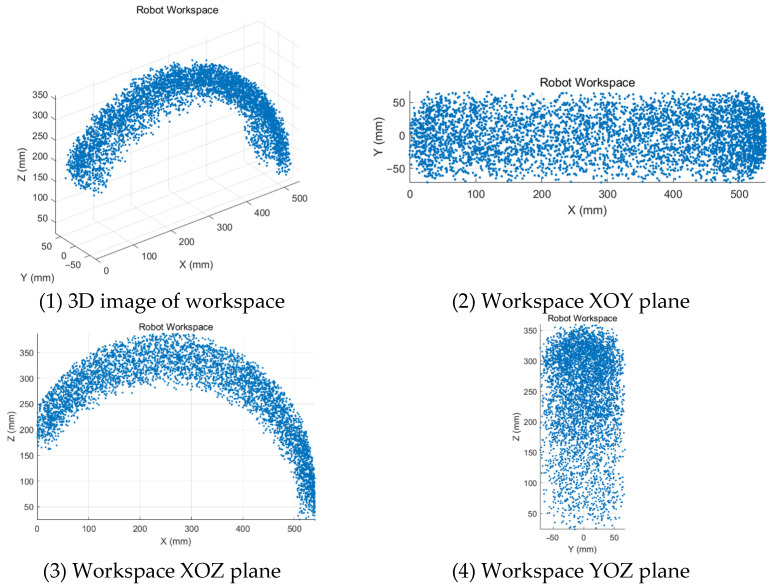
Workspace of the upper limb exoskeleton system.

Within the given range of joint angles, a simulation analysis software was used to simulate random values, and 5000 random joint angles were generated in each joint space. The set of generated random points is the robot’s workspace, and the three-dimensional and two-dimensional cloud maps of the workspace of the robot are established, as shown in Figure 5. The resulting 3D workspace resembles a toroidal volume segment that closely matches the natural range of motion of the human upper limb for rehabilitation tasks. From the workspace point cloud map, it can be seen that the workspace of the exoskeleton is sufficiently large and dense, and the workspace adequately covers typical elbow and wrist motions required in clinical recovery exercises, such as flexion–extension and internal–external rotation.

### 2.3. The Control System Based on the Angle Sensor

The apparatus employs an MPU6050 angle sensor (InvenSense, San Jose, CA, USA)to gather and analyze signals related to the positioning and orientation of the healthy limbs. The MPU6050 delivers highly accurate data for axial acceleration and angular velocity across all axes, offering quick responsiveness, streamlined data processing, and energy-efficient design. The MPU6050, a commonly used six-axis attitude sensor from InvenSense, integrates a three-axis accelerometer and a three-axis gyroscope. It supports I2C communication and has an acceleration measurement range of ±2 g to ±16 g. The gyroscope had a range of ±250/s to ±2000/s, with sensitivities of 16384 LSB/g (at ±2 g) and 131 LSB/(s) (±250/s). The MPU6050 sensor exhibits significant advantages in upper-limb exoskeleton rehabilitation training systems because of its high-precision angle measurement capability, low power consumption, miniaturized design, real-time responsiveness, ease of integration, compatibility, stability, interference resistance, and cost-effectiveness. Its integrated three-axis accelerometer and three-axis gyroscope accurately measure the static angle and dynamic angular velocity of joints, ensuring precise control and real-time adjustment during movement. Furthermore, the MPU6050’s low-power design renders it suitable for portable or wearable devices, thereby reducing the burden of wear and enhancing comfort. Given that the exoskeleton operates with two degrees of freedom, the system selects data from MPU6050 that aligns with the joint movement axis and transmits it to the STM32 control board for further processing. Upper-limb rehabilitation exoskeleton robots typically utilize various drive mechanisms, including motor, hydraulic, pneumatic, and tendon sheath drives [37]. Among these, motor drives are most prevalent in exoskeleton systems. This setup incorporates two stepper motors and a servo to power the exoskeleton robot. The 4248 stepper motor (KHMOS, Shenzhen, China) is a two-phase hybrid stepper motor according to the NEMA 17 standard, with a step angle of 1.8 and a holding torque of approximately 0.6 to 1.2 N m. A 4248-stepper motor is utilized to control the forearm’s telescopic movement, another 4248-stepper motor manages the telescopic slide table, and an MG995 servo governs the wrist’s internal and external rotation. The MG995 servo (TowerPro, Shenzhen, China) supports a voltage range from 4.8 V to 7.2 V, capable of delivering up to 10 kg·cm of torque at 6 V. It features a metal gear structure for stability and is widely used in robotic joints and steering systems. The servo operates on a standard PWM signal (50 Hz), offering fast response times with a response time of 0.13 s/60 at 6 V, making it ideal for applications requiring precise rotation angles and torque.

The upper limb exoskeleton rehabilitation training system employs two complementary control methods to accommodate different rehabilitation needs and stages. The first method is based on a lower-level control system that manages continuous passive rehabilitation exercises for an impaired limb. This method is especially effective in the early stages of rehabilitation or when the patient is unable to autonomously control the limb. Through the lower-level control system, the device can provide continuous, passive movement assistance according to a preset program, helping the patient maintain joint flexibility and range of motion without requiring active participation. This passive movement helps prevent joint stiffness, muscle atrophy, and other issues, promotes blood circulation, and lays the foundation for subsequent active rehabilitation exercises.

The second method involves the use of an angle sensor to monitor the position and orientation data of a healthy limb in real time, which then guides the impaired limb to perform synchronized movements. The angle sensor was installed on a healthy limb and could precisely capture every angle change in the limb. These data were then transmitted in real time to the exoskeleton control system. Based on these data, the exoskeleton device adjusts the movement of the impaired limb to ensure that it moves in synchrony with the healthy limb. The key advantage of this method is its ability to enable active synchronized movements, helping the patient gradually regain the ability to perform active movements while enhancing neural plasticity. This approach contributes to improving motor coordination and functional recovery.

**Figure 6:**
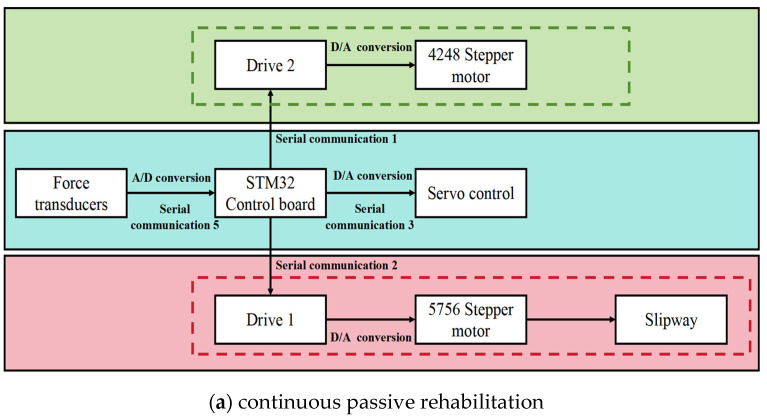

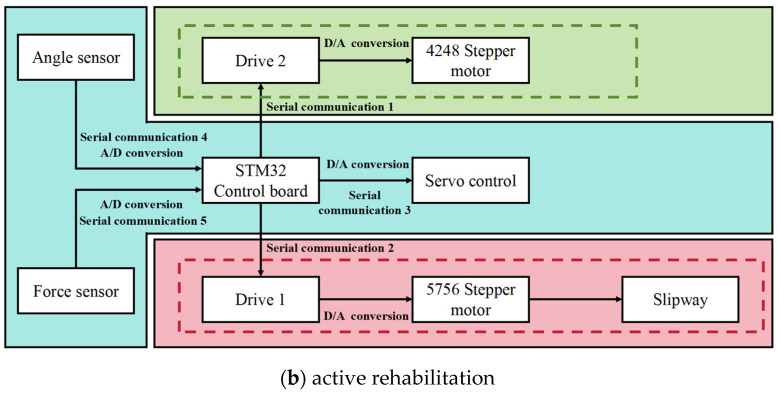
Control system block diagrams of two rehabilitation training modes.

Figure 6 illustrates the block diagrams of these two control systems and clearly shows their working principles and complementary relationships. The lower-level control system provides continuous passive movement support to the impaired limb through mechanical transmission and dynamic models, whereas the angle sensor system achieves synchronized control between the healthy and impaired limbs through real-time monitoring and data transmission. The combination of these two systems forms a flexible and efficient rehabilitation training model that can dynamically adjust movement patterns and training intensity based on the patient’s rehabilitation progress and needs, thus providing a personalized rehabilitation program. Video S1 in the Supplementary Materials shows the dynamic process of the upper-limb exoskeleton system under active and passive rehabilitation modes. This system not only improves rehabilitation efficiency, but also offers a more precise and comfortable rehabilitation experience for the patient.

The control algorithm for continuous passive and active rehabilitation is described in detail below:

The control algorithm of active rehabilitation is to realize the periodic reciprocating motion of the affected limb within the range of $\left[\theta_{\min},\theta_{\max}\right]$ by setting the interval of angular change and step length. The core algorithm is as follows:

**Step 1:** Angle state update formula

(4) $$\theta_{t+1}=\theta_{t}+s_{t}\cdot \Delta \theta$$

where θ_t_ represents the target angle at the current moment; Δθ represents the angle step length (unit angle change); st ∈ {−1, 1} represents the direction state; if the boundary is reached, it is reversed; and t is the time step.

**Step 2:** Boundary judgment and direction reversal

(5) $$s_{t+1}=\left\{\begin{array}{l} -1,\;\mathrm{if}\;\theta_{t}\geq \theta_{max}\\ 1,\;\mathrm{if}\;\theta_{t}\leq \theta_{min}\\ s_{t},\mathrm{otherwise} \end{array}\right.$$

**Step 3:** Motor control output (position mode):

(6) $$u_{t}=\theta_{t}$$

where u_t_ is the target angle-setting value of the motor controller.

Continuous passive control algorithm: This algorithm drives the motor on the affected side to complete synchronous movements by reading the motion angle of the healthy limb as a reference signal in real-time.

**Step 1:** Healthy limb angle acquisition (IMU)

(7) $$\theta^{H}\left(t\right)=f\mathrm{IMU}\left(a_{t},\omega_{t}\right)$$

where θ^H^ (t) represents the angle of the healthy limb, a_t_ and ω_t_ represent the acceleration and angular velocity of the MPU6050 sensor at the current moment; f_IMU_ is the attitude solution function.

**Step 2:** Synchronize control instruction output:

(8) $$u_{t}=\theta^{H}\left(t\right)$$

In other words, the target of the controller is the target angle that the affected side should reach at the current moment.

## 3. Results

### 3.1. Experimental Evaluation of the Upper Limb Exoskeletons Rehabilitation Training System

**Figure 7:**
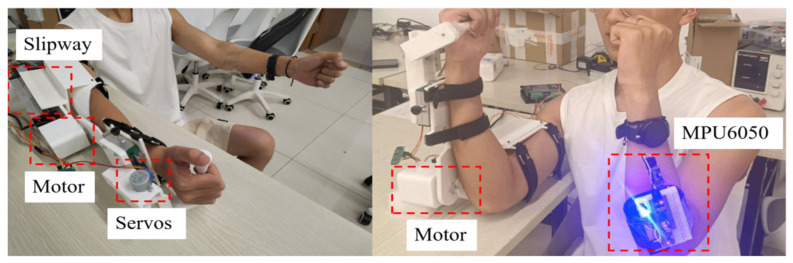
The experimental setup of the upper limb exoskeleton rehabilitation training system.

Figure 7 depicts a comprehensive upper-limb exoskeleton rehabilitation robot and rehabilitation training experiment. During the study, researchers positioned the robot and conducted ongoing passive rehabilitation training assessments and exercise rehabilitation experiments. The rotation of the elbow, wrist, and sliding table was evaluated along with a motor protection mechanism. An angle sensor was employed to continuously monitor the arm rotation by comparing the data from the affected limb to that of the healthy limb. The angle discrepancy was calculated to evaluate the device’s performance. Additionally, during active training tests, when the healthy limb’s angle exceeded a predetermined threshold, the device’s protective feature was activated, halting the movement of the affected limb. The affected limb remained stationary until the healthy limb returned to the same angle. Subsequently, the affected limb resumed synchronization with the healthy limb within the established threshold range.

To ensure that the MPU6050 Angle sensor provided accurate and reliable data in the experiment, we carried out standardized installation and calibration operations on the angle sensor before the experiment. First, the sensor should be fixed to the rigid parts of the limb and exoskeleton structure to ensure that the posture changes can truly reflect the joint movement without being disturbed by loosening or structural vibrations. After the installation is complete, the sensor needs to be calibrated statically and dynamically, including zero-bias correction of the accelerometer and gyroscope, gravity direction identification, and three-axis alignment. In the calibration process, a standard initialization program is used to read the initial offset through the interface library provided by the sensor manufacturer, and the Kalman filter algorithm is used for attitude estimation optimization. In addition, to avoid interference from temperature drift and other factors, the calibration should be completed at a constant temperature or stable environment, and the sensor should be kept in a stable working state.

### 3.2. The Continuous Passive Rehabilitation Training Experiments

**Figure 8:**
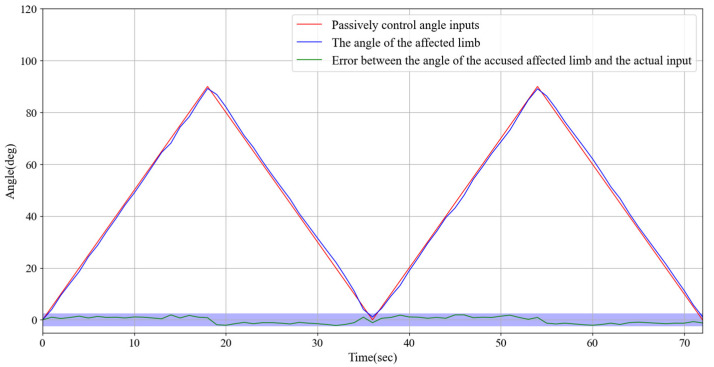
Experimental test results of continuous passive rehabilitation training and the error between the angle of the affected limb and the actual input.

To achieve precise real-time monitoring of the rotation angle of the impaired limb, we utilized a high-precision angle sensor with the monitored angle data displayed on a screen for easy observation and recording. During the flexion and extension tests, the angle range was set from 0° to 90° to ensure that the test results covered the common range of limb movements. In the experimental design, the program-preset angle was used as the control group to provide a reference for the ideal movement trajectory, whereas the actual angle displayed by the impaired limb during movement was defined as the experimental group, serving as the key data source for validating the performance of the exoskeleton device. During the experiment, the exoskeleton motor, through precise torque control and speed adjustment, effectively guided the impaired limb to move stably along a predetermined angle trajectory, demonstrating excellent follow-through performance. Figure 8 presents the specific test results, which confirm the high stability and controllability of the exoskeleton system. In addition, by adjusting the rotational speed of the motor, the system can accommodate the individual needs of different patients and provide flexible rehabilitation training schemes. For instance, training can start with low-speed assistance and gradually increase the range of motion and speed to promote the functional recovery of the impaired limb.

### 3.3. The Synchronous Exercise Rehabilitation Training Experiment

High-precision angle sensors were installed on both impaired and healthy limbs to continuously monitor and record their movement angles. The angle data were displayed on a screen in real time to facilitate observation and comparison. During the experiment, we set an angle range of 0–90° for the flexion and extension tests, ensuring that the tests covered the main range of limb movement. In the experimental design, the angle data of the healthy limb were used as a control group to provide an ideal reference for the movement trajectory, while the angle data of the impaired limb were used as the experimental variable to verify the ability of the device to guide the movement of the impaired limb in real time.

**Figure 9:**
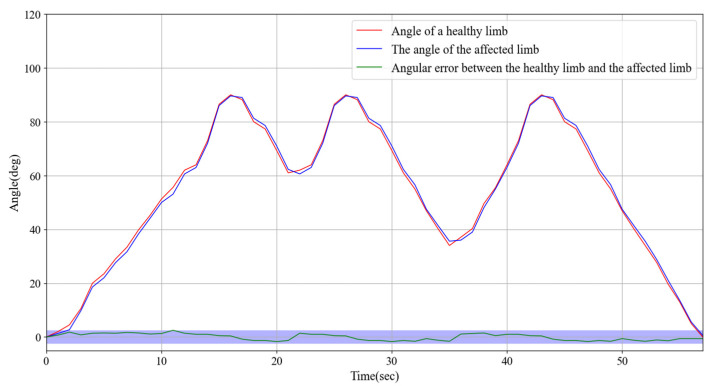
Experimental test of synchronous exercise rehabilitation training and the angular error between the healthy limb and the affected limb.

The experiment specifically tested rotational movements within the 0° to 90° range, with detailed recordings of the angle changes and dynamic responses of both impaired and healthy limbs. As shown in Figure 9, the results demonstrated that the impaired limb exhibited excellent responsiveness, quickly following the motion trajectory of the healthy limb. The angle discrepancy between the two limbs remained consistently within 2.5°, indicating a high degree of synchronization and accuracy. Furthermore, although there was a slight delay caused by the wireless signal transmission from the healthy limb, the response time of the impaired limb did not exceed 0.2 s. According to the experimental data in Figure 9, during the synchronous movement, when the impaired limb reaches the same angle as the healthy limb, the time that the healthy limb reaches the angle minus the impaired limb reaches the angle, and the measured delay time is within 0.2 s. For an angular error of 2.5, we conducted an error analysis after the experiment. Primarily, signal transmission delay and interference can induce latency in the wireless signal, affecting the synchronization of the impaired limb and increasing the error fluctuations. In addition, sensor accuracy and measurement noise, such as zero-point drift and transmission error, may contribute to the instability of angular data. Concurrently, the physiological properties of the limb, including muscle fatigue and variations in joint flexibility, can result in angular fluctuations during movement, particularly when the coordination of the impaired limb is compromised. The control system response of the exoskeleton device may also exhibit delayed or adaptive issues, and the instability in the driving system may lead to transient errors. Environmental factors such as temperature variations can also influence sensor performance, resulting in angular data fluctuations. Furthermore, the individual physiological state and the complexity of movement patterns can impact the stability of movement, especially during high-velocity or complex movements, where sensors may encounter difficulties in capturing rapidly changing angles, thereby increasing the error. The entire rotation process was smooth and consistent with no noticeable jitter or lag.

These findings suggest that the angle sensors and wireless transmission technology used in this system effectively support synchronized limb movements and real-time monitoring capabilities. Moreover, they demonstrated the potential application of the system in limb rehabilitation training, thereby providing a strong foundation for further optimization and application in personalized rehabilitation solutions.

### 3.4. Investigation of the Root Mean Square Error in Angle Measurement for Synchronous Motion Control in Upper Limb Exoskeleton Rehabilitation Training Systems

The primary objective of this experiment was to evaluate the angular error in the synchronous motion control of the upper-limb exoskeleton rehabilitation system, with particular emphasis on the synchronization discrepancies between healthy and affected limbs. As the rehabilitation process progresses, the patient’s motor function and the adaptability of the exoskeleton system may evolve. Consequently, the experiment was designed to assess the performance of the system at various stages of multiple training sessions. To ensure that the results were representative and scientifically valid, 20 subjects(non-patient) were selected as research subjects, encompassing a range of medical conditions and rehabilitation requirements.

**Figure 10:**
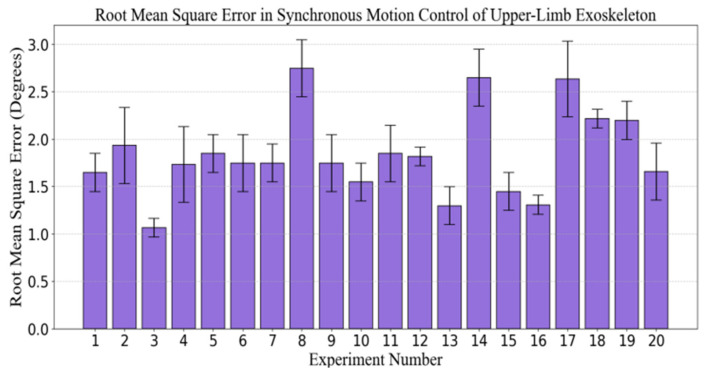
Angle root mean square error (RMSE) experiment of synchronous motion control for the upper limb exoskeleton rehabilitation training system.

Each subject participated in multiple rehabilitation training sessions during which angular errors between the healthy and affected limbs were recorded. Specifically, the experiment monitored the angular difference between the healthy and affected limbs driven by the exoskeleton system in real time to analyze the precision of the system in synchronous motion control. After each training session, all angular error data were collected and organized, and the root mean square error (RMSE) was calculated (as shown in Figure 10) for each subject during different training sessions.

Statistical analysis of the experimental results indicated that the angular error fluctuated across the different experimental sequences. The majority of the errors were concentrated between 1.00 and 1.50°, suggesting that the exoskeleton system effectively simulated the movement of the healthy limb while maintaining high synchronization precision. However, the data also revealed fluctuations in the error during certain training sessions, reflecting some instability in the system under specific conditions. The maximum error occurred in the 8th experiment, approaching 2.5°, while the minimum error occurred in the 3rd experiment, approaching 0.75°.

This phenomenon may be attributed to factors such as the subject’s physiological condition, adaptability of the exoskeleton system, and intensity of the training regimen, indicating that the exoskeleton system demonstrates high precision and stability in synchronous motion control, effectively facilitating rehabilitation training. Despite minor fluctuations in errors during individual experiments, the majority of training sessions maintained angular errors within an acceptable range, thus demonstrating the system’s reliability and efficacy at various stages of training. Further research could investigate the adaptability of the system across a more diverse patient population and its long-term performance to optimize the effectiveness of exoskeleton-assisted rehabilitation training.

### 3.5. Experiments on Synchronous Rehabilitation Training Under Different Load Protection Thresholds

Stroke rehabilitation training is typically conducted in multiple stages, aiming to gradually expand the range of motion as the patient’s recovery progresses, helping to restore limb function. In the early stages of rehabilitation, patients’ mobility is limited, and they can only exercise within a small range of motion. As rehabilitation advances, range of motion gradually increases, allowing for more diversified movements. To help patients navigate this progressive rehabilitation process, rehabilitation devices typically adjust the threshold values to control the range of motion and provide appropriate assistance at different stages.

In this study, training conditions were evaluated across different threshold ranges, with several staged ranges set, including 0–15°, 0–30°, 0–45°, 0–60°, 0–75°, and 0–90°, to simulate various rehabilitation stages. During the operation, when the movement of the healthy limb exceeds the set threshold, the rehabilitation device immediately activates a safety mechanism to prevent excessive movement of the impaired limb, thereby avoiding injury caused by uncoordinated motion. Specifically, the movement of the impaired limb halts immediately, maintaining its current angle, and the impaired limb will only resume synchronized movement with the healthy limb once it returns to the same angle.

**Figure 11:**
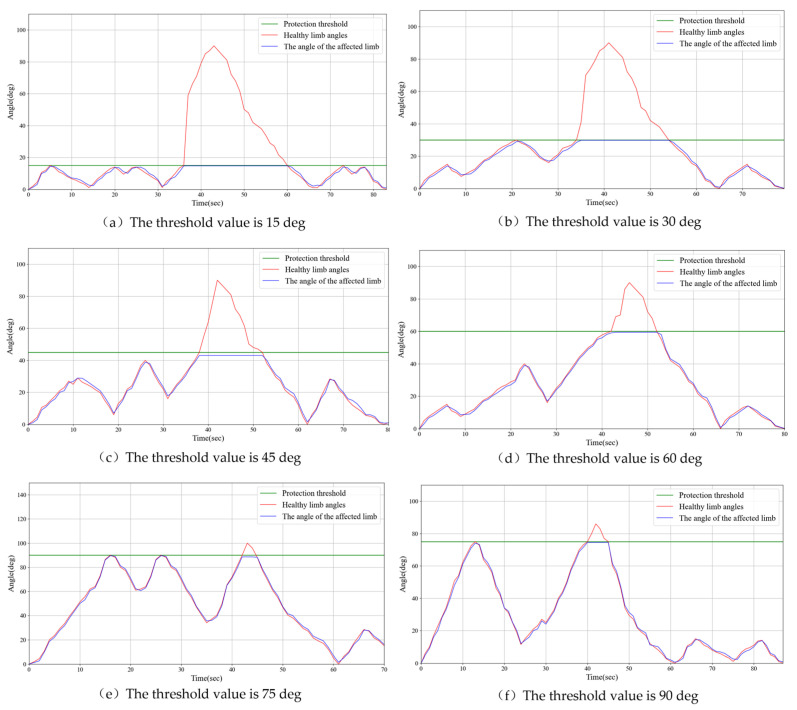
Examples of experimental results for threshold protection in rehabilitation training.

Figure 11 illustrates the experimental results. The experiments showed that when the healthy limb exceeded the set threshold, the impaired limb triggered the protective mechanism within 0.2 s, automatically halting its movement and maintaining a stable angle. From the experimental data in Figure 11, during the synchronized movement, the impaired limb triggers the protective mechanism. The delay time measured, within 0.2 s, is the point at which the angle of the impaired limb begins to remain constant, subtracted from the angle at which the healthy limb reaches the set maximum threshold. The timely response of this mechanism effectively prevents unnecessary pulling or pressure on the impaired limb, thus avoiding injury. Once the healthy limb was realigned with the angle of the impaired limb, the impaired limb resumed synchronized movement with the healthy limb and continued rehabilitation training.

The results of this study Indicate that by setting appropriate threshold ranges and implementing timely safety mechanisms, rehabilitation devices can effectively guide patients through safe movement training, as the range of motion gradually increases. This progressive training approach not only helps patients gradually increase limb flexibility and strength during recovery, but also ensures safety during the training process, preventing secondary injuries caused by excessive or uncoordinated movements. Furthermore, the real-time monitoring and response mechanisms of the device provide personalized rehabilitation support, allowing patients to adjust exercises based on their recovery progress. This provides new insights into stroke rehabilitation and offers valuable references for the future optimization and design of rehabilitation devices.

### 3.6. Experiments on Synchronous Rehabilitation Training Under Different Loads

**Figure 12:**
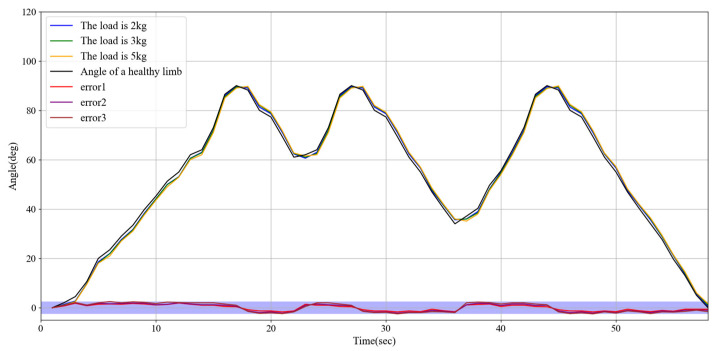
Experimental results on synchronous rehabilitation training under different loads.

This experiment investigates the performance of the upper limb exoskeleton robot in synchronous movement under varying loads due to the differing limb weights of patients. This study aims to evaluate whether a robot can maintain adequate movement synchronization and accuracy under diverse load conditions. Different load conditions were established at the slave end: 2 kg (light), 3 kg (medium), and 5 kg (and heavy). Synchronous motion tests were conducted for each condition. Figure 12 illustrates the experimental results, demonstrating that the exoskeleton robot exhibits satisfactory adaptability under various loading conditions. The angle error for both the healthy limb and the affected limb remains within 2.5 degrees.

### 3.7. Experiments on Rehabilitation Training of Synchronous Motor at Different Speeds

**Figure 13:**
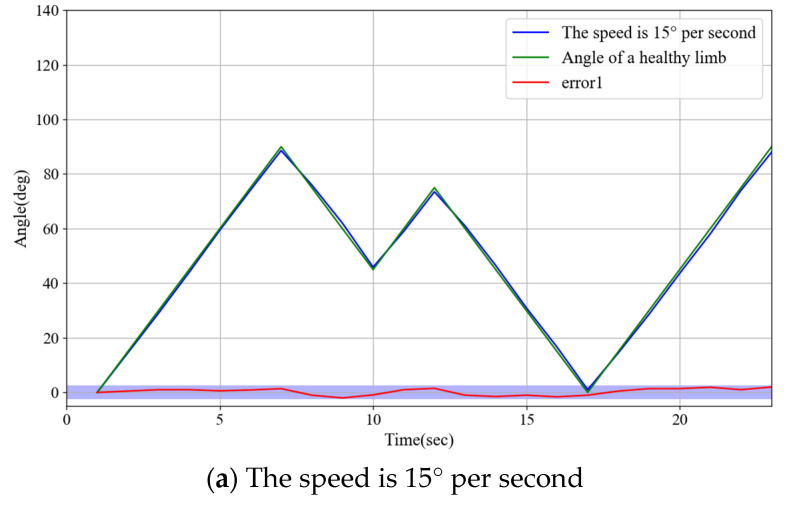

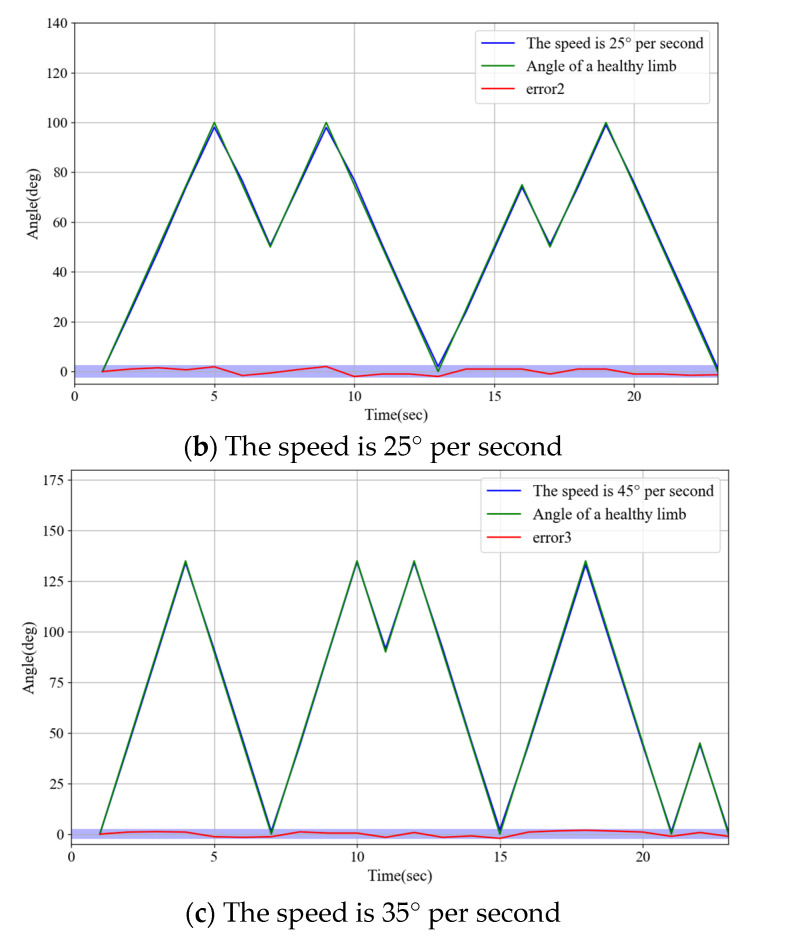
Experimental results on rehabilitation training of synchronous motor at different speeds.

During the actual rehabilitation process, the movement speed of the unaffected limbs is controlled by the patient’s volitional control. The objective of this experiment was to investigate whether variations in speed could influence the responsiveness of the robot control system to synchronous movements of both limbs and to ensure the safety of rehabilitation training. The experiment established three speed settings for unaffected limb movement: low speed at 15°/s, suitable for gentle rehabilitation training; medium speed at 25°/s, appropriate for moderate-intensity functional recovery exercises; and high speed at 45°/s, conducive to improving movement coordination and flexibility. The synchronization error between both limbs was evaluated by recording angular changes in the unaffected and affected limbs. The experimental results, as illustrated in Figure 13, demonstrate that the robot can maintain high synchronization and stability under various speed conditions.

## 4. Discussion

Experiments demonstrated that the system exhibited high precision in continuous passive rehabilitation and synchronous motor rehabilitation training. As illustrated in Figure 8, the exoskeleton system effectively guided the affected limb along a predetermined trajectory, with the angular deviation consistently maintained within an acceptable range. Similarly, Figure 9 illustrates the high synchronization accuracy of the system in synchronous motor rehabilitation training, with the angular error between the healthy limb and the affected limb consistently below 2.5° with minimal response delay. These results indicate that the system is suitable for real-time monitoring and control, and can accurately guide the movement of the affected limb. The consistency and reliability of the system were further corroborated by the root mean square error (RMSE) analysis (Figure 10). Despite minor factors, such as sensor noise, patient physiological condition, and system adaptability, the error was predominantly maintained within the 1.0–1.5° range, reflecting the stability and robustness of the control mechanism. The experimental evaluation of exercise safety protection mechanisms (see Figure 11) underscores the importance of integrating safety functions in rehabilitation devices. The system demonstrated the ability to halt the affected limb movement within 0.2 s when the healthy limb exceeded the threshold, indicating its capacity to effectively prevent excessive extension and injury. This adaptive mechanism is particularly crucial in the early stages of rehabilitation, when patients have limited motor control capacity. Furthermore, the system exhibited excellent adaptability under varying load conditions (Figure 12). Even with load changes (2–5 kg range), the synchronization error remained stable below 2.5°, indicating that the exoskeleton can accommodate the needs of diverse patients. The performance of the system under different speed conditions (see Figure 13) further validates its responsiveness and stability in low-, medium-, and high-speed scenarios, ensuring safety and efficiency at different stages of rehabilitation. Despite the excellent performance of the system in terms of precision and adaptability, several limitations persist. Signal delay and measurement error: Wireless signal transmission introduces a slight delay (up to 0.1 s) that, although minimal in most scenarios, can affect fast motion synchronization. Moreover, the sensor noise and zero-point drift occasionally cause angular fluctuations. Additionally, individual patient differences in physiological factors, such as muscle fatigue, joint flexibility, and variations in coordination, can influence the system performance during high-speed or complex movements.

Studies have demonstrated that the upper limb exoskeleton system is capable of providing individualized solutions according to patient requirements at various stages of rehabilitation. The system exhibited adaptability to different speeds, loads, and ranges of motion, highlighting its versatility and suitability for diverse medical conditions. Moreover, the incorporation of safety mechanisms ensures that the device can be securely utilized in both clinical and domestic environments. To further enhance system performance, future research should prioritize the development of high-precision sensors to mitigate the impact of noise and environmental interference. The introduction of machine learning algorithms to analyze patient-specific data would enable the dynamic adjustment of training parameters, facilitating personalized rehabilitation. Additionally, conducting longitudinal studies would allow for the evaluation of the effectiveness and durability of the system during extended rehabilitation cycles. Finally, optimizing the signal transmission protocol reduces latency and improves real-time response capabilities.

## 5. Conclusions

This study focused on the mechanical structure design, development of the control system, and relevant experimental tests of the rehabilitation training system, with the aim of addressing the requirements of the upper limb exoskeleton during rehabilitation. In accordance with the specific rehabilitation needs of diverse patients, the system incorporates two rehabilitation modes: active rehabilitation training mode and passive rehabilitation training mode. The active rehabilitation mode is appropriate for patients with a degree of autonomous movement capability. In this mode, patients can utilize their muscle strength in conjunction with the exoskeleton system. For patients with diminished or complete absence of autonomous motor ability, the passive rehabilitation training mode is more suitable. Both approaches have demonstrated promising outcomes in various experimental evaluations. The experimental results indicated that the upper-limb exoskeleton rehabilitation training system exhibited stable and rapid response characteristics. When the unaffected healthy limb exceeds the threshold, the affected limb activates the protective mechanism within 0.2 s and maintains the current angle. Once the angles of both limbs are equivalent, the affected limb resumes the synchronous movement. The affected limb demonstrated a rapid response, with an angular difference of less than 2.5°, reflecting high synchrony with unaffected healthy limb movements. The proposed rehabilitation training system for upper limb exoskeleton rehabilitation demonstrates significant potential to enhance the personalization, safety, and efficiency of rehabilitation treatment, providing an innovative rehabilitation solution for patients.

## Figures and Tables

**Table 1:** D-H parameters.

Joint (i)	α (i)	a (i)	d (i)	θ (i)	Joint Variables	Variable Scope
1	0	0	0	0	/	/
2	90°	0	*d*_2_	*θ*_2_	*d*_2_/*θ*_2_	250~350 mm/0~150°
3	−90°	0	0	0	/	/
4	90°	0	*d*_4_	*θ*_4_	*d*_4_/*θ*_4_	190~240 mm/ −70°~90°

## Data Availability

Data are contained within the article.

## Supplementary Materials

The following supporting information can be downloaded at https://www.mdpi.com/article/10.3390/s25133984/s1, Video S1: Operation under active and passive rehabilitation modes.
